# Evaluation of selected hematological, biochemical and oxidative stress parameters in stored canine CPDA-1 whole blood

**DOI:** 10.1186/s12917-022-03353-x

**Published:** 2022-07-01

**Authors:** Jolanta Bujok, Eliza Wajman, Natalia Trochanowska-Pauk, Tomasz Walski

**Affiliations:** 1 Department of Animal Physiology and Biostructure, Division of Animal Physiology, Wrocław University of Environmental and Life Sciences, C.K. Norwida 31, 50-375 Wrocław, Poland; 2 Department of Biomedical Engineering, Faculty of Fundamental Problems of Technology, Wrocław University of Science and Technology, Wybrzeże Wyspiańskiego 27, 50-370 Wrocław, Poland; 3 Department of Physics and Biophysics, Wrocław University of Environmental and Life Sciences, C.K. Norwida 25, 50-375 Wrocław, Poland

**Keywords:** Red blood cells, Blood storage, Storage lesions, Oxidative stress, Free hemoglobin

## Abstract

Blood transfusions are mainly given to intensive care patients; therefore, additional complications that could arise from storage lesions in preserved blood should be avoided. It has been shown that human stored red blood cells are subject to changes that are considered to be a number of interdependent processes involving metabolic disarrangement and oxidative stress. The aim of our study was to determine alterations in selected hematological and biochemical parameters and to assess whether and when oxidative stress is a significant phenomenon in stored dog CPDA-1 whole blood. Ten ½ unit bags of whole blood donated from dogs and preserved with CPDA-1 (anticoagulant containing citrate, phosphate, dextrose and adenine) were stored for 5 weeks. Each week, a 9 ml sample was drawn aseptically to measure hematological parameters, selected metabolites, free hemoglobin content, osmotic fragility, antioxidant enzyme activity, total antioxidant capacity, malondialdehyde concentration and protein carbonyl content.

The results revealed an MCV decrease in the first week of storage and then a gradual increase; osmotic fragility decreased at that time and remained low throughout the study period. Leukodepletion became significant in the fourth week of storage. The free hemoglobin concentration continuously increased, with the greatest changes observed in the last two weeks of storage. The total antioxidant capacity changed in a reverse manner. Superoxide dismutase and glutathione peroxidase activities decreased from week 0 to week 3, and catalase activity tended to decrease over time. The highest malondialdehyde concentrations in blood supernatant were measured in the first week of storage, and the carbonyl concentration increased after 35 days.

Hematological changes and oxidative stress are already present in the first week of storage, resulting in depletion of the antioxidant system and subsequent accumulation of oxidation products as well as erythrocyte hemolysis, which are most pronounced at the end of the storage period.

## Background

Treatment with blood products is becoming a routine therapeutic procedure in veterinary medicine. The demand for blood from donors is constantly increasing. Thus, there is growing interest in the quality control of the administered blood products. This is especially true because in most cases, blood transfusions are received by intensive care patients, who are at increased risk of developing multiple organ damage [1]. Therefore, additional complications that could arise from storage lesions in preserved blood should be avoided. Several human studies have shown that ‘old’ stored blood can increase mortality in critically ill patients [2, 3]. These findings were confirmed in an experimental model of canine pneumonia in which blood that was stored for 42 days and administered to animals increased the risk of mortality compared to blood stored for only seven days [4]. The release of free iron from old damaged erythrocytes was postulated to be one of the main mechanisms of toxicity [5]. A retrospective study in canine patients also showed that transfusions of ‘older’ blood were associated with coagulopathies and increased the risk of mortality in patients with immune-mediated hemolytic anemia [6].

According to human studies, when stored under refrigerated conditions, red blood cells (RBCs) are subject to changes that are considered to constitute a number of interdependent processes. Metabolism is not fully inhibited, with possible dysregulation due to the altered environment. This may both lead to and result from a limited antioxidant defense system. Typically, depletion in ATP and 2,3-DPG with potassium leakage occur in stored erythrocytes. These and other changes in metabolic pathways may further translate into accumulation of oxidative damage of lipids and proteins, depletion of antioxidants and potentially irreversible changes in the structure and function of the cytoskeleton, plasma membrane and enzymes [7–9]. When in circulation again, these cells can be either subject to rapid clearance or may induce endothelial dysfunction, damage and coagulopathies [10–13]. Which of these changes is most significant for potential adverse reactions and whether oxidative damage has an important role need to be determined.

Canine blood banking is mainly based on guidelines from human medicine, but there are some differences between the cells, especially in terms of volume regulation and the content of antioxidant enzymes [14]. Some ex vivo aging processes have been studied in dog blood, with results indicating release of proinflammatory cytokines and microparticles, changes in erythrocyte volume, potassium release and ATP and 2,3-DPG depletion, along with other biochemical alterations [9, 15–18]. It was also shown that the presence of leukocytes may partially perpetuate several metabolic disarrangements in canine preserved blood [19–21]. Nevertheless, it remains unclear how fast redox system imbalances occur and to what extent oxidative stress correlates with other ‘storage lesions’. Therefore, the objective of our study was to characterize oxidative stress together with hematological and biochemical parameters in CPDA-1-stored dog blood.

## Results

### Hematological parameters

**Fig. 1:**
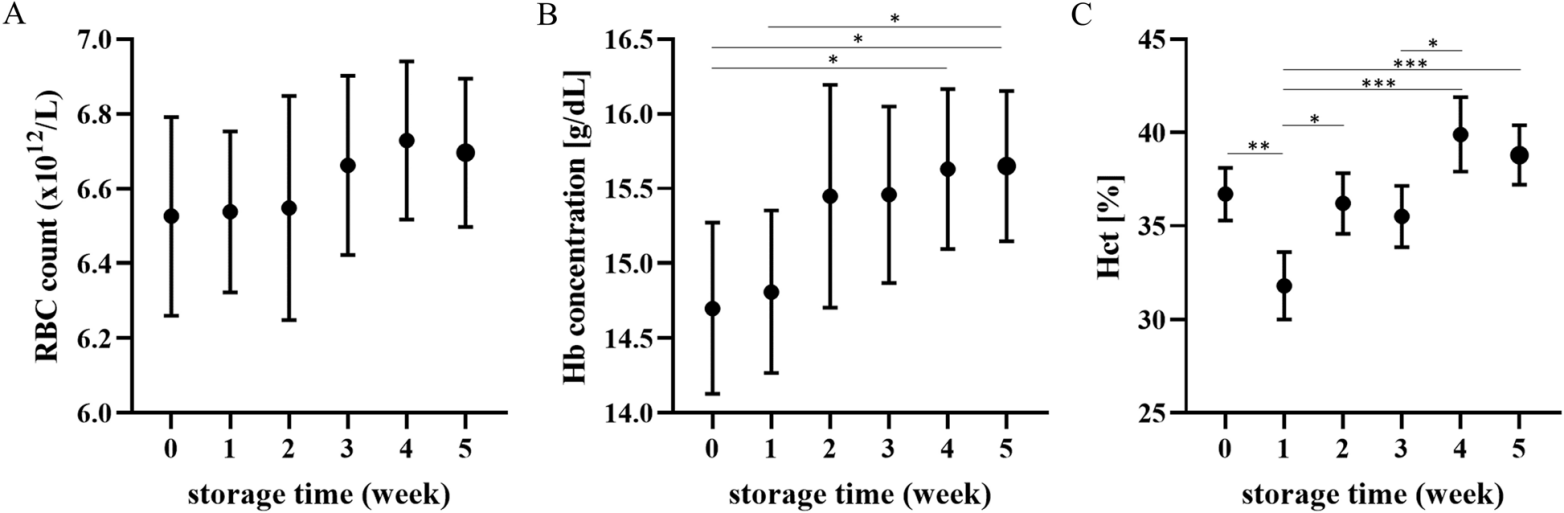
Weekly changes in red blood cell (RBC) count (**A**), hemoglobin (Hb) concentration (**B**), and hematocrit (Hct) (**C**) in canine CPDA-1 whole blood stored for five weeks. Data are presented as the mean ± s.e.m.; number of measurements, *n* = 10. Significant differences are marked with asterisks. * *p* < 0.05, ** *p* < 0.01, *** *p* < 0.001

**Fig. 2:**
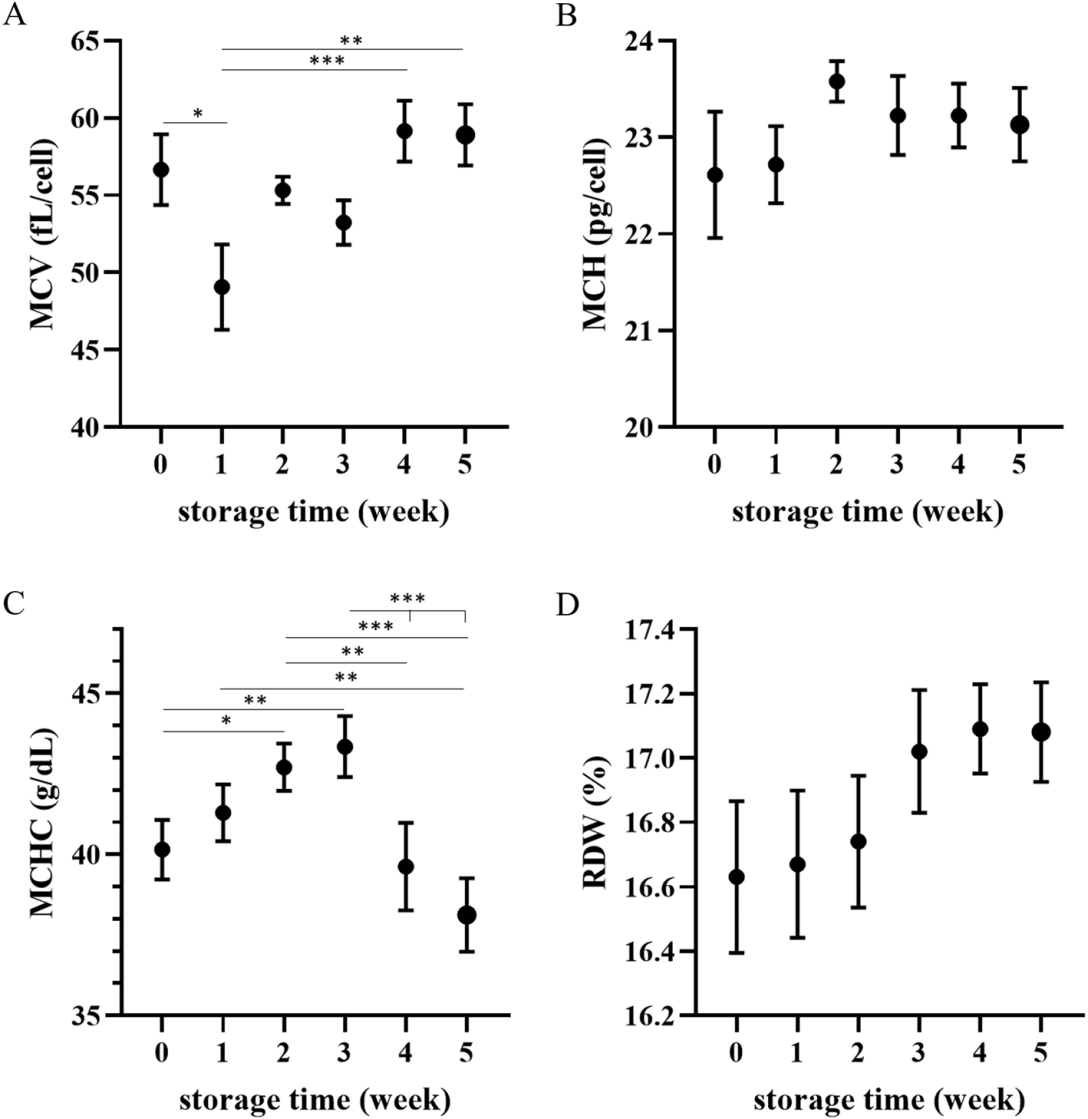
Weekly changes in (**A**) mean corpuscular volume (MCV), (**B**) mean corpuscular hemoglobin (MCH), (**C**) mean corpuscular hemoglobin concentration (MCHC), and (**D**) red blood cell distribution width (RDW) in dog CPDA-1 whole blood stored for five weeks. Data are presented as the mean ± s.e.m.; number of measurements, *n* = 10. Significant differences are marked with asterisks. * *p* < 0.05, ** *p* < 0.01, *** *p* < 0.001

**Fig. 3:**
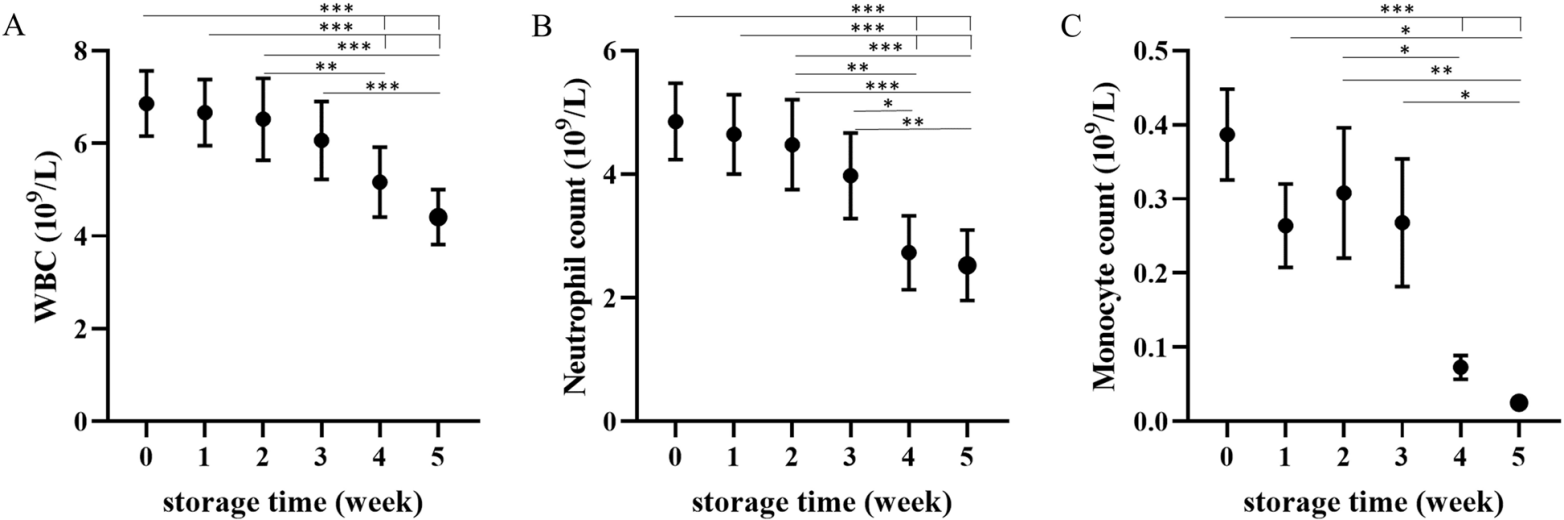
Weekly changes in (**A**) white blood cell count (WBC), (**B**) neutrophil count, and (**C**) monocyte count in dog CPDA-1 whole blood stored for five weeks. Data are presented as the mean ± s.e.m.; number of measurements, *n* = 10. Significant differences are marked with asterisks. * *p* < 0.05, ** *p* < 0.01, *** *p* < 0.001

Hematocrit (Hct) decreased in the first week of storage (*p* < 0.01) and then gradually increased (*p* < 0.05 or less); hemoglobin (Hb) concentration also increased toward the end of storage (*p* < 0.05 at Week 0 vs. Weeks 4,5, and W1 vs. W5), though RBCs did not change significantly. Mean corpuscular volume (MCV) followed the changes in Hct, being lower in the first week of storage (*p* < 0.05) and then gradually increasing until the end of the experiment (*p* < 0.01 or less at W1 vs. W4 and W5) (Figs. 1 and 2). In addition, mean corpuscular hemoglobin concentration (MCHC) increased in the second and third weeks of storage (*p* < 0.05 and *p* < 0.01, respectively), followed by a decrease (*p* < 0.01 or less at W2 vs. W4, W5 and *p* < 0.001 at W3 vs. W4, W5). Red blood cell distribution width (RDW) tended to increase over time, though this effect was not significant. Leukodepletion was significant in the fourth week of storage (*p* < 0.01 or less at W0-W2 vs. W4, W5; *p* < 0.001 at W3 vs. W5), mainly due to the decrease in the number of phagocytes (Fig. 3).

### Free hemoglobin and osmotic fragility

**Fig. 4:**
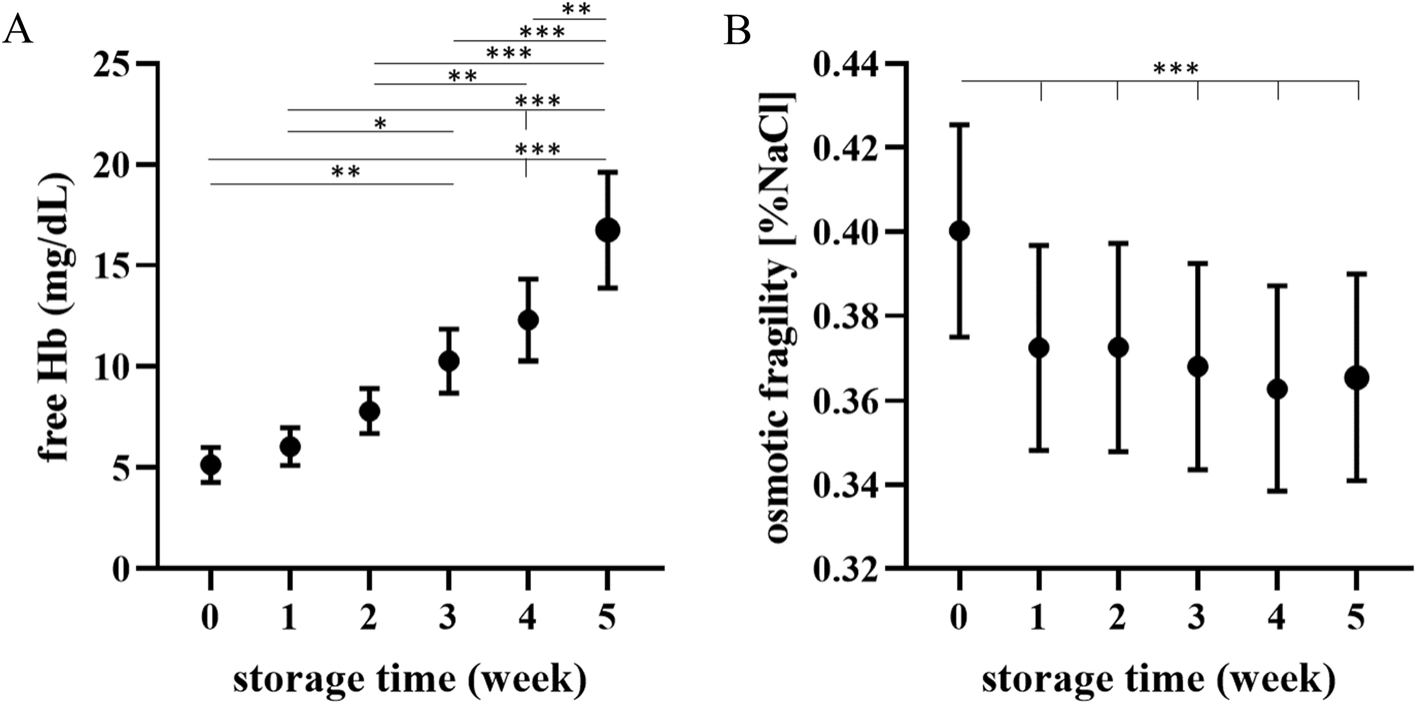
Weekly changes in (**A**) free hemoglobin (Hb) concentration and (**B**) osmotic fragility of red blood cells in dog CPDA-1 whole blood stored for five weeks. Data are presented as the mean ± s.e.m.; number of measurements, *n* = 10. Significant differences are marked with asterisks. * *p* < 0.05, ** *p* < 0.01, *** *p* < 0.001

The concentration of free Hb in supernatant increased gradually throughout the experimental period (*p* < 0.05 or less at W0-W1 vs. W3-W5; *p* < 0.01 or less at W2 vs. W4,W5). Spontaneous hemolysis varied greatly among individuals; however, a steady increase in free Hb was a consistent feature of all preparations (Fig. 4a). Osmotic fragility decreased in the first week of storage and remained low for the rest of the study (*p* < 0.001 at W0 vs. W1-W5) (Fig. 4b).

### Glucose and lactate

**Fig. 5:**
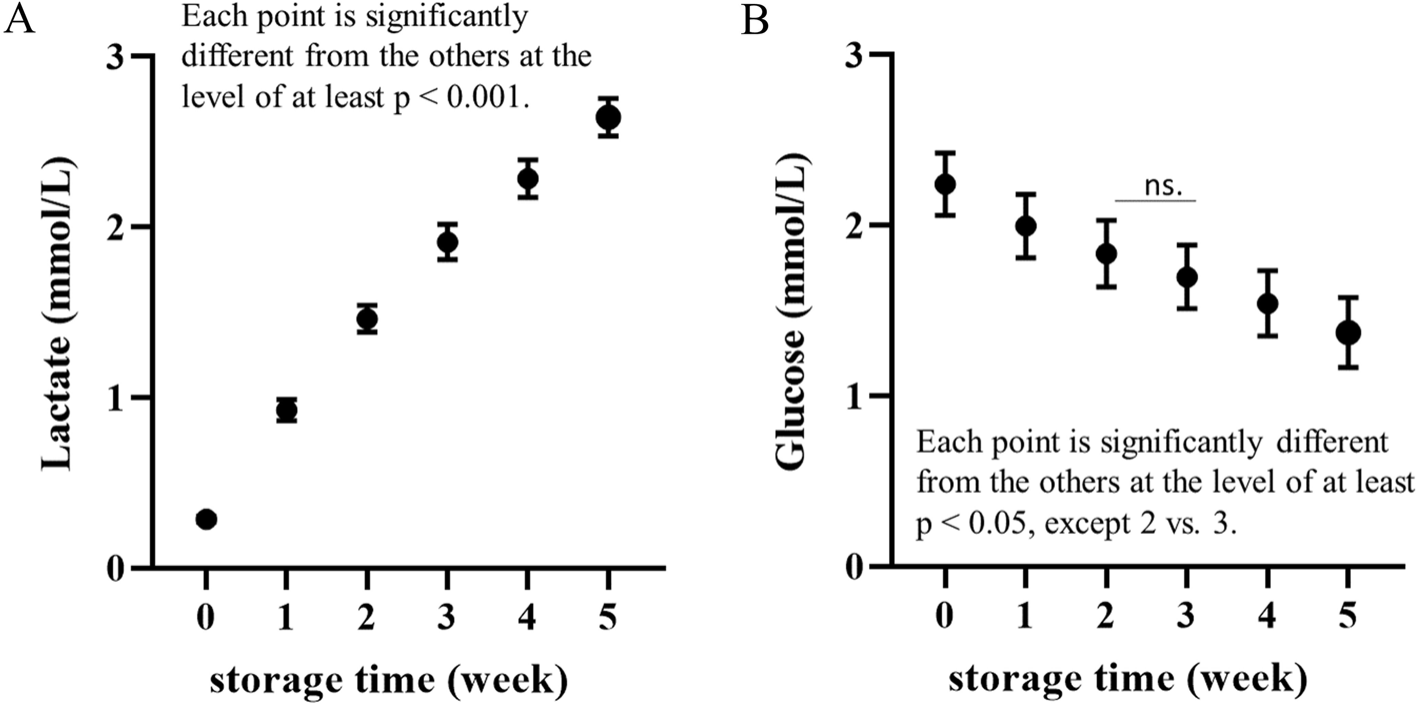
Weekly changes in (**A**) glucose and (**B**) lactate concentrations in dog CPDA-1 whole blood stored for five weeks. Data are presented as the mean ± s.e.m.; number of measurements, *n* = 10

The concentration of glucose in supernatant was higher than the physiological concentration throughout the storage period; however, a nearby linear decrease occurred week by week (each point was significantly different from the others at a level of at least *p* < 0.05 except W2 vs. W3). A reduction in glucose concentration was associated with lactate generation, the concentration of which exceeded normal values on the seventh day of storage and continued to increase until the 35th day of the experiment (each point was significantly different from the others at the level of at least *p* < 0.001) (Fig. 5).

### Antioxidant enzymes and total antioxidant capacity

**Fig. 6:**
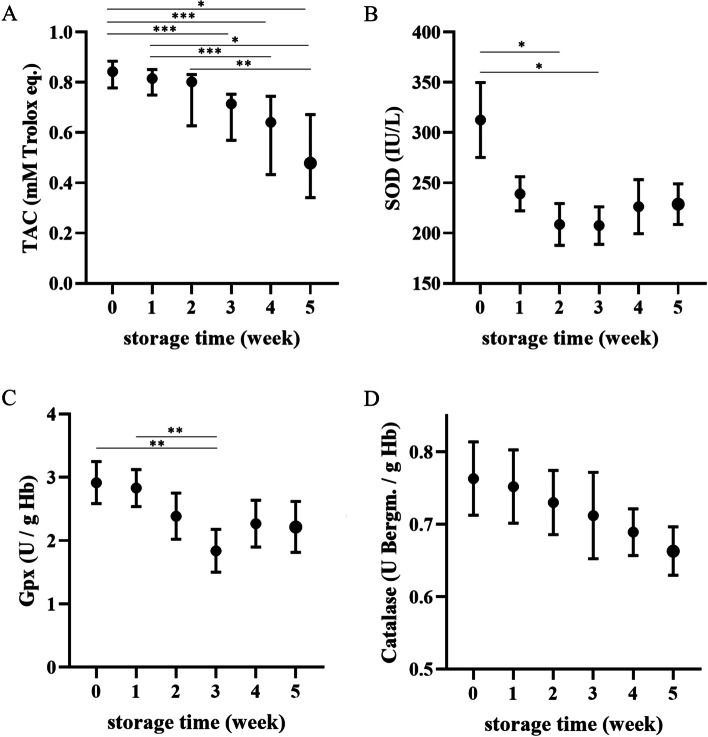
Weekly changes in (**A**) total antioxidant capacity (TAC) in blood supernatant and in erythrocyte antioxidant enzyme activities, (**B**) superoxide dismutase (SOD), (**C**) glutathione peroxidase (GPx), and (**C**) catalase (CAT), in canine CPDA-1 whole blood stored for five weeks. TAC data are presented as the median ± interquartile range, and others are presented as the mean ± s.e.m.; number of measurements, *n* = 10. Significant differences are marked with asterisks. * *p* < 0.05, ** *p* < 0.01, *** *p* < 0.001

The total antioxidant capacity (TAC) significantly decreased from the third week of storage (*p* < 0.001 at W0 vs. W3) and reached the lowest values in the last week of the experiment (*p* < 0.05 or less for W0-W2 vs. W5) (Fig. 6). Superoxide dismutase (SOD) activity started to decrease in the first week of storage and was significantly lower in the third and fourth weeks (*p* < 0.05 at both time points) compared to Day 0. Similarly, glutathione peroxidase (GPx) activity was lower in the third week of storage than on the day of collection and in Week 1 (*p* < 0.01 for both time points). Catalase (CAT) tended to decrease over time; however, the change was not significant.

### Oxidation products

**Fig. 7:**
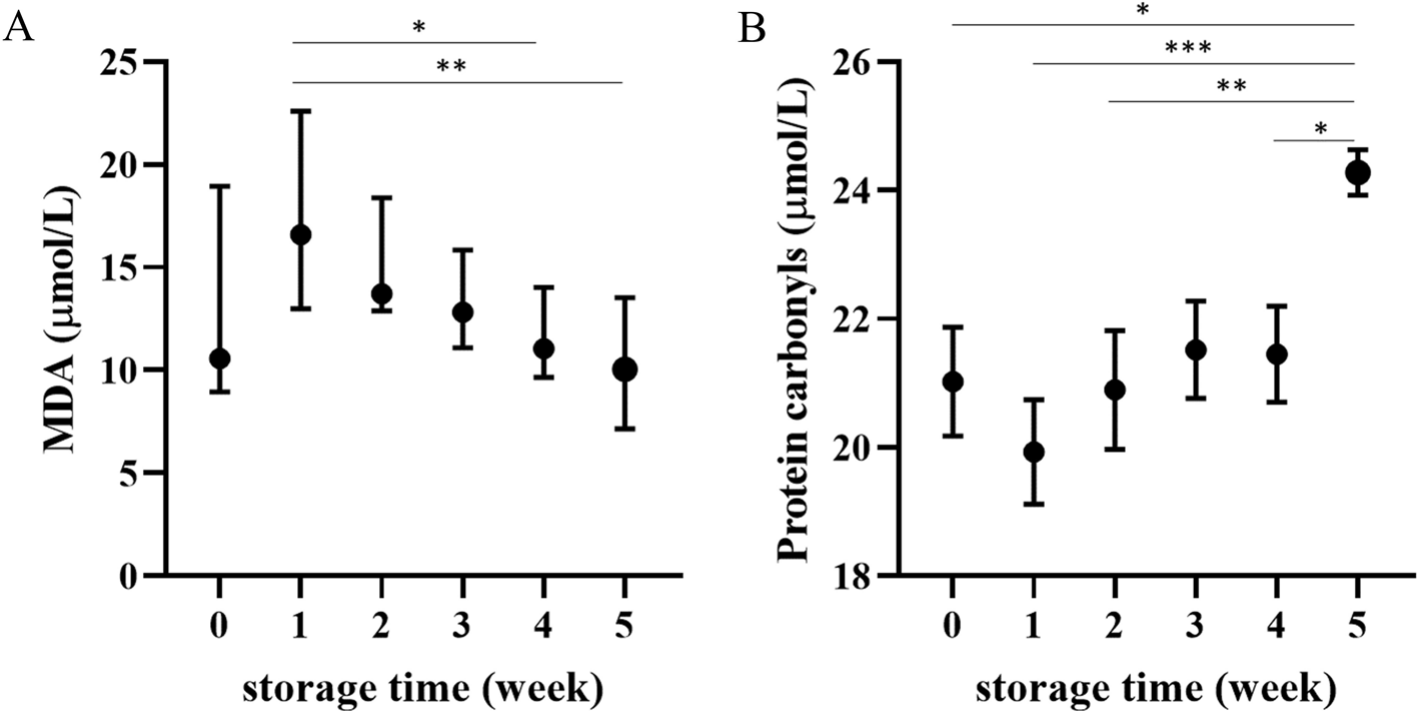
Weekly changes in (**A**) malondialdehyde (MDA) concentration and (**B**) protein carbonyl concentration in the supernatant of canine CPDA-1 whole blood stored for five weeks. Data of the MDA concentration are presented as the median ± interquartile range, and protein carbonyls are presented as the mean ± s.e.m.; number of measurements, *n* = 10. Significant differences are marked with asterisks. * *p* < 0.05, ** *p* < 0.01, *** *p* < 0.001

The highest concentrations of malondialdehyde (MDA) were observed on the seventh day of storage, which then gradually decreased to significantly lower values in the last two weeks of the study (*p* < 0.05 and *p* < 0.01 at W1 vs. W4, W5, respectively). However, MDA was generally quite high throughout storage. The protein carbonyl concentration in supernatant increased only in the fifth week of storage (*p* < 0.05 or less at W0-W2 and W4 vs. W5) (Fig. 7).

## Discussion

We observed significant changes in hematological and biochemical parameters as well as the occurrence and dynamics of oxidative stress in canine CPDA-1-treated blood. Alterations in hematological parameters were evident from the first week of storage and together with a decrease in osmotic fragility suggest dehydration of the cells with subsequent loss of volume or shape-maintaining ability. Human erythrocytes typically swell during storage, which is thought to be at least in part the result of Na^+^/K^+^ ATPase inhibition under cold storage conditions. The size change is reversed to that seen in vivo in aging erythrocytes, which become dehydrated due to Ca^2+^-activated K^+^-channel activation. Citrate in the preservation fluid chelates calcium and therefore probably inhibits cell dehydration [7]. However, the heterogenicity of erythrocytes increases with the formation of echinocytes and microcytes [22]. Dog erythrocytes lack Na^+^/K^+^-ATPase, and excluding some Asian breeds, they are low-potassium cells. In our study, cells decreased in size in the first week of storage and then gradually regained volume. Our results are consistent with the findings of Antognoni et al. (2021) [15], who reported a decrease in erythrocyte MCV in the first weeks of storage regardless of leukocyte content and then a gradual increase in this parameter. Donor RBCs are placed under hyperosmolar conditions, though CPDA-1 solution has a higher osmolarity (470 mOsm/l) than the normal dog plasma osmolarity, which most likely induces primary water loss [23]. Over the next weeks of storage, the MCV of cells increases, which might be associated with equilibration to higher osmolarity. This corresponds to our results, which show that cell osmotic fragility decreases in the first week of storage and then remains similar for the ensuing weeks. Similar changes in osmotic fragility were also seen in a study on canine leukoreduced and nonleukoreduced whole blood and packed RBCs [19]. Although Stefani et al. observed that potassium concentration increases significantly between Days 0 and 7 of storage, whether this ion plays a role in early volume changes in canine stored erythrocytes remains to be elucidated [19]. An increase in cell size can also be the result of a generalized alteration in cation transporter function due to cold storage conditions and acidification. Dog erythrocytes primarily regulate their volume by sodium outflow through the Na^+^-Ca^2+^ exchanger, the function of which is dependent on active calcium transport [24]. Inhibition of this regulatory mechanism may be expected during storage at 4 °C because both ATP depletion and pH decrease are observed in stored dog blood [9, 19]. Nonetheless, the exact role of cation channels in dog erythrocyte volume alterations during storage has not yet been reported.

Dog erythrocytes stored in CPDA-1 are subjected to long-term hyperglycemic conditions. According to our study, the glucose concentration remain largely above normal values throughout the study period, even though anerobic glucose metabolism does not cease, which results in continuous lactate formation and thus acidification of the environment. This is consistent with studies on dog and human stored blood [9, 19, 25, 26]. Whether hyperglycemia itself is a lesion-provoking factor remains unresolved. According to some studies, the concentration of glycated Hb in donated blood increases over time. It is not known whether this has a significant impact on metabolism and cytoskeleton remodeling [27]. Overall, acidification of the cell environment may alter membrane transport and metabolism, leading to faster erythrocyte deterioration [28].

Free Hb is considered one of the major signs of ‘storage lesions’ in blood. Similar to other studies on stored dog erythrocytes, an increase in free Hb was observed throughout storage in our study [29]. However, the most significant changes were observed in the last two weeks of the study, suggesting an avalanche-like nature of this process. Corresponding results have been described for canine packed RBCs [30]. This may be related to free iron release, which is known to act as an oxidant, and further oxidation of molecules important for maintaining cell integrity, including Hb and cytoskeleton proteins [31, 32]. Free Hb release correlated well with a decrease in TAC in our study, indicating depletion of antioxidant defense mechanisms with the progression of hemolysis in the erythrocyte environment. The RBC metabolism rate is decreased under refrigerated conditions, followed by a failure to reduce oxidized Hb and depletion of reduced glutathione [7]. Superoxide anions formed during Hb autooxidation are first converted by SOD to H_2_O_2_, which is then neutralized by GPx and CAT [33]. In our study, SOD and GPx activities decreased by week 3 of storage, which preceded an increased rate of hemolysis and faster loss of TAC in the supernatant. This suggests that the oxidative stress in CPDA-1 canine whole blood is present early during storage and leads to significant antioxidant system depletion in erythrocytes within 21 days, with a subsequent increase in oxidation product accumulation and erythrocyte lysis. In general, hemolysis in stored blood may be associated with an increase in intravascular heme in recipients, which causes endothelial injury, inflammation and complement activation, as well as in nontranferrin-bound iron overload leading to facilitation of bacterial proliferation [6, 34–36]. Both may result in adverse events in critically ill patients. Klein (2017) proved experimentally in a canine pneumonia model that “old” blood transfusion is associated with higher mortality, whereby a significant increase in nontransferrin-bound iron and cell-free Hb followed by hypertension were observed due to the NO-scavenging action of Hb and pulmonary necrosis [5]. On the other hand, it was stated that this vasoactive action of released Hb may be beneficial in patients in hemorrhagic shock [5].

The supernatant MDA concentration in our study was significantly higher in the first week of storage than in the fourth and fifth weeks, and these high concentrations of MDA were accompanied by a decrease in SOD activity in erythrocytes. On the one hand, this suggests that most of the polyunsaturated lipid peroxidation of plasma occurs during the first week of storage, with subsequent consumption of antioxidant enzymes. On the other hand, an increase in MDA in plasma may also be associated with platelet activation and thromboxane A_2_ (TXA_2_) synthesis. It was shown that MDA is a byproduct of platelet thromboxane synthase [37]. In human whole blood and platelet concentrates, unstimulated platelet activation increases significantly by Day 7 of storage, and the ability of thrombocytes to be activated decreases after 21 days [38]. Our results may be associated with this phenomenon because we measured the highest concentration of MDA after one week of storage and it was significantly higher than after four or five weeks. Comparison with platelet-deprived blood products would help to explain the source of MDA in CPDA-1-stored whole blood supernatant. The increase in MDA may also be linked to neutrophil activation and ROS generation, with subsequent oxidation of polyunsaturated lipids, as leukocyte activation in stored canine blood products has been described [17, 21]. Studies on human blood products also suggest that early MDA formation may be linked to leukocytes or platelets because the MDA concentration in stored human erythrocytes was shown to increase over time, whereas that measured in the supernatant of RBC concentrates was unchanged [39, 40]. In contrast, MDA in supernatant and erythrocytes increased until the 19th day of storage and then remained unchanged in a study on whole human blood [41]. Moreover, prestorage leukoreduction decreases oxylipid formation in stored RBCs; however, it does not eliminate this process [42]. The MDA generated has the ability to bind to biomolecules such as proteins, with the epsilon amino group of lysine being the main target, and the reaction is facilitated at lower pH values [43]. During cold storage, lactate is generated, and blood pH decreases; thus, the formation of MDA-protein adducts may be intensified toward the end of “shelf life” [9, 19]. The extent and consequences of MDA adduct formation have yet to be elucidated.

In our study, the supernatant carbonyl concentration increased only at 5 weeks after blood collection. According to studies conducted on human stored erythrocytes, carbonylation processes begin shortly after collection, and the concentration of carbonyls in the erythrocyte membrane and cytoskeleton proteins increases over time; however, a decrease is observed after Day 28 [44, 45]. This coincides with an increase in carbonyl content in the microvesicles that form during the storage period. As proposed by Delobel et al. (2012) [46], proteins subjected to carbonylation may be repaired to some extent by the proteasome, though further accumulation of carbonylation products leads to protein aggregation and loss of functionality. Proteasome activity decreases as well, and the clustered oxidated proteins can no longer be unfolded for repair. Erythrocytes may then expel the aggregates of damaged proteins through a vesiculation process. Moreover, these cells will be more likely to undergo hemolysis and release into the plasma not only Hb but also other carbonylated proteins. Regardless, the increase in carbonyl concentration in the supernatant did not correlate well with the progress of hemolysis in our study, suggesting that microvesicles may be the main source of carbonyls. One of the carbonylated proteins may be the band 3 protein. Carbonylation of this protein is believed to be a step in the formation of antigens for autoantibodies recognizing senescent and damaged erythrocytes [46, 47]. If carbonyls appear in the plasma of canine blood in the 5th week of storage, oxidative damage to band 3 and other proteins may occur in erythrocytes stored for three to four weeks. In our study, antioxidative enzyme activity decreased at week 3 of storage, suggesting their depletion under pro-oxidative conditions. Cells with protein oxidative damage may be cleared from circulation directly after transfusion. Moreover, it has been postulated that microvesicles from stored erythrocytes not only promote coagulation and phagocytosis but are also immunologically active [46, 47]. Nevertheless, the extent of carbonylation of erythrocyte proteins in stored dog blood remains to be determined, which is a limitation of our study.

## Conclusion

In conclusion, significant metabolic and hematological alterations together with evidence of oxidative stress were observed in CPDA-1 canine whole blood as early as after seven days of storage. The most pronounced changes, such as significant hemolysis, depletion of total antioxidants and presence of protein oxidation products, were evident in the fifth week of storage and were preceded by erythrocyte antioxidant enzyme consumption. In general, early changes may have a significant impact on the morbidity and survival of patients and may depend on the health status of the recipient. CPDA-1 blood stored for more than 28 days is characterized by more irreversible changes and probably should be avoided, especially for patients with sepsis, SIRS, and severe hemolytic anemia in which a proinflammatory state and oxidative stress are already pronounced [5, 6, 48].

## Materials and methods

### Animals and blood collection

Blood was obtained from 10 volunteer dogs that were engaged as donors in veterinary clinics. The animals met the donor criteria of the animal blood bank; that is, they were clinically healthy, vaccinated and regularly receiving antiparasitic drugs and did not travel abroad. Each owner signed an informed consent form to use the blood for research. The dogs were 4–8 years old (mean 6.0 ± 0.4) and weighed 28 to 62 kg (mean 43.0 ± 4.2). Two of the dogs were males; all animals were neutered. Blood was collected aseptically from the cephalic vein into ½ unit sterile bags with approx. 32 ml of CPDA-1 anticoagulant (citrate, phosphate, dextrose, adenine; Ravimed Sp. z o.o., Łajski, Poland) designed for blood storage for 35 days. The blood was gently mixed during collection and then immediately placed in a disinfected laboratory refrigerator set at 4 °C (CHL2/ZLN 85 COMF, POL-EKO Aparatura Sp.j., Wodzislaw, Poland). The blood was kept at 4 °C for 5 weeks (35 days). On Days 0, 7, 14, 21, 28 and 35, 9 ml samples were collected after gentle mixing through an attached needle-free valve (Safeflow, BBraun Melsungen AG, Melsungen, Germany), which was cleaned with an alcohol swab before and after use to prevent bacterial contamination. The blood was transferred to a 1 ml fluoride oxalate tube, 1 ml EDTA-K tube, three heparinized capillaries, and three 2 ml Eppendorf tubes; the remaining blood was used to measure osmotic fragility. The blood in the Eppendorf tubes was centrifuged for 10 min at 4000 x g and 4 °C. The supernatant containing the plasma with CPDA and the erythrocyte sediment were each collected into four separate Eppendorf tubes and immediately frozen at − 80 °C until analysis.

### Hematological parameters

One milliliter of blood was transferred to an EDTA-K probe and analyzed by an automated analyzer (VetScan HM5 Hematology Analyzer, Abaxis, USA). Furthermore, three heparinized capillaries were filled with blood and centrifuged at 20,000 x g for 5 min to directly measure Hct values. RBC indices, including MCV, mean corpuscular hemoglobin (MCH), and MCHC, were calculated from RBC and Hb levels measured using the analyzer, and Hct was determined via the capillary method. RDW was measured by an automated analyzer.

### Hemoglobin concentration measurement

The Hb concentration was measured spectrophotometrically at 540 nm using the Drabkin method (Hb reagent, Randox Ltd., Ireland). Cell-free supernatant and RBC sediment were obtained by centrifuging the blood samples for 10 min at 1750 × g. An aliquot of 5 μl of the supernatant (free Hb measurement) or RBC sediment was added to 1.25 ml of reagent and incubated for 3 min. Absorbance was measured at 540 nm (Nicolet Evolution 60, Thermo Scientific, USA), and the concentration was calculated from the standard curve (free Hb) or by multiplying the obtained absorbance by the calibration value of 36.77 (Hb concentration). Each sample was measured in triplicate [49, 50].

### Hemolysis curve and determination of osmotic fragility

Erythrocytes for the OF test were isolated by centrifugation at 1750 × g for 10 min at 4 °C, washed three times in phosphate-buffered saline (PBS; pH = 7.4) and diluted in PBS to obtain 40% Hct. The OF curve was generated as previously described [49, 50]. The RBC samples were placed in a series of tubes containing different concentrations of sodium chloride solution from isotonic to low ionic strength close to that of distilled water (0–145 mM NaCl buffered by 10 mM phosphate buffer, pH = 7.4); the suspension was centrifuged at 1750 × g for 4 min after 30 min. The obtained supernatant was examined spectrophotometrically (Nicolet Evolution 60, Thermo Scientific, USA). The amount of Hb released, proportional to the number of lysed cells, was estimated by colorimetric analysis at 540 nm. The absorbance values were normalized using (1), that is, 0% - no hemolysis occurred and 100% - all cells were hemolyzed:

1 $${\mathrm{A}}_{\mathrm{N}}=\frac{{\mathrm{A}}_{\mathrm{x}}-{\mathrm{A}}_{\mathrm{iso}}}{{\mathrm{A}}_{\mathrm{aq}}-{\mathrm{A}}_{\mathrm{iso}}}100\%$$

where A_N_ is the normalized relative absorbance, A_x_ is the absorbance of the solution for the measured sample, A_iso_ is the absorbance of the solution in an isotonic medium, and A_aq_ is the absorbance when 100% of the cells are hemolyzed in distilled water. The concentration of NaCl solution when 50% of the cells are hemolyzed is a measure of the OF.

### Glucose and lactate

One milliliter of blood from each collection was transferred to a fluoride oxalate tube and kept cool until analysis using an automated biochemistry analyzer (Cobas, Roche, Germany).

### Antioxidant enzyme activity

SOD in erythrocytes was measured spectrophotometrically using a method based on inhibition of the xanthine and xanthine oxidase reaction leading to the production of red formazan dye with a commercial reagent kit (RANSOD, Randox Ltd., Ireland). Then, 0.25 ml of erythrocyte sediment was hemolyzed in 1.75 ml of double distilled water and briefly centrifuged to spin the debris; 20 μl of the supernatant was diluted in 2.5 ml of sample diluent to achieve a reaction inhibition rate of 30–60%. Each sample was measured in triplicate, and SOD activity was calculated from a standard curve.

GPx activity in erythrocytes was determined by a method based on that of Paglia and Valentine [51] using a commercial kit (RANSEL, Randox Ltd., Ireland). First, 0.05 ml of erythrocyte sediment was diluted in 3 ml of diluent agent; after mixing the diluted sample with cumene and reagent, the decrease in absorbance at 340 nm was measured for 2 min.

CAT activity in erythrocytes was measured spectrophotometrically by the Aebi method [52]. Erythrocyte sediment was diluted 1:1 in double-distilled water. Next, 0.05 ml of this erythrocyte suspension was dissolved in 0.425 ml of double distilled water. After 15 min, 0.03 ml of the supernatant was added to 0.87 ml of KH_2_PO_4_/Na_2_PO_4_ buffer. The reaction was initiated with 30% H_2_O_2_ in KH_2_PO_4_ buffer. Absorbance was assessed every 15 seconds for 30 sec. CAT activity in Bergmayer units was calculated from the following formula:

2 $$\mathrm{CT}\;\left[\mathrm U\;\mathrm{Bergm}.\right]=\frac{\ln{\mathrm A}_0}{\ln{\mathrm A}_{30}}\cdot\mathrm a\cdot0.481$$

### Total antioxidant capacity

TAC was measured spectrophotometrically by a method based on inhibition of 2,2-azino-bis(ethylbenzene-thiazoline-6-sulfonic acid) (ABTS) oxidation by metmyoglobin to a free radical that is green–blue in color and absorbs light at 750 nm. A commercial kit was used (Antioxidant Assay Kit, Cayman Chemicals, Arbor, USA). The TAC in undiluted samples was calculated from the standard curve for Trolox, and the result is expressed in Trolox milliequivalents.

### Oxidation products

MDA was measured spectroscopically in supernatant by a method based on the reaction of lipid peroxidation products with thiobarbituric acid at 100 °C after protein precipitation in trichloroacetic acid (TCA) using a commercial kit (TBARS (TCA method) Assay Kit, Cayman Chemicals, An Arbor, USA). Absorbance at 535 nm was measured, and the concentration of MDA was calculated from a standard curve.

Protein carbonyls in supernatant were determined by a method based on the reaction of 2,4-dinitrophenylhydrazine with carbonyls, resulting in the formation of a Schiff base for the production of the corresponding hydrazone absorbing light at 360–385 nm. Samples were diluted four times in HPLC-grade water, and carbonyls were determined using a commercial kit (Protein Carbonyl Colorimetric Assay Kit, Cayman Chemicals, An Arbor, USA).

### Statistical analysis

Calculations were performed using the statistical package STATISTICA 13.3 (StatSoft, Inc., USA). The normal distribution hypothesis was tested with Kolmogorov–Smirnov and Shapiro–Wilk tests. One-way analysis of variance (ANOVA) with repeated measures followed by Tukey’s post-hoc test or Friedman’s ANOVA followed by Dunn’s post-hoc test was performed to determine significant changes in the measured parameters during storage. Differences between means were considered significant when *p* < 0.05.

## Data Availability

The data that support the findings of this study are available from the corresponding author [J.B.] upon reasonable request.

## Abbreviations

2,3-DPG: 2,3-diphosphoglycerate
ABTS: 2,2-azino-bis(ethylbenzene-thiazoline-6-sulfonic acid)
ATP: Adenosine triphosphate
CAT: Catalase
CPDA-1: Citrate phosphate dextrose adenine
EDTA-K: Ethylenediaminetetraacetic acid potassium salt
GPx: Glutathione peroxidase
Hb: Hemoglobin
Hct: Hematocrit
MCH: Mean corpuscular hemoglobin
MCHC: Mean corpuscular hemoglobin concentration
MCV: Mean corpuscular volume
MDA: Malondialdehyde
OF: Osmotic fragility
RBC: Red blood cell
RDW: Red blood cell distribution width
SOD: Superoxide dismutase
TAC: Total antioxidant capacity
TCA: Trichloroacetic acid
WBC: White blood cell
